# New Perspectives on Iron Uptake in Eukaryotes

**DOI:** 10.3389/fmolb.2018.00097

**Published:** 2018-11-19

**Authors:** Harry G. Sherman, Carolyn Jovanovic, Snow Stolnik, Kim Baronian, Alison J. Downard, Frankie J. Rawson

**Affiliations:** ^1^Division of Regenerative Medicine and Cellular Therapies, School of Pharmacy, University of Nottingham, Nottingham, United Kingdom; ^2^Walgreens Boots Alliance, Nottingham, United Kingdom; ^3^Division of Molecular Therapeutics and Formulation, School of Pharmacy, University of Nottingham, Nottingham, United Kingdom; ^4^School of Biological Sciences, University of Canterbury, Christchurch, New Zealand; ^5^MacDiarmid Institute for Advanced Materials and Nanotechnology, School of Physical and Chemical Sciences, University of Canterbury, Christchurch, New Zealand

**Keywords:** iron, transplasma membrane electron transport systems (tPMETS), plasma membrane oxidoreductase system, redox, non-transferrin bound iron, electron transfer, transferrin

## Abstract

All eukaryotic organisms require iron to function. Malfunctions within iron homeostasis have a range of physiological consequences, and can lead to the development of pathological conditions that can result in an excess of non-transferrin bound iron (NTBI). Despite extensive understanding of iron homeostasis, the links between the “macroscopic” transport of iron across biological barriers (cellular membranes) and the chemistry of redox changes that drive these processes still needs elucidating. This review draws conclusions from the current literature, and describes some of the underlying biophysical and biochemical processes that occur in iron homeostasis. By first taking a broad view of iron uptake within the gut and subsequent delivery to tissues, in addition to describing the transferrin and non-transferrin mediated components of these processes, we provide a base of knowledge from which we further explore NTBI uptake. We provide concise up-to-date information of the transplasma electron transport systems (tPMETSs) involved within NTBI uptake, and highlight how these systems are not only involved within NTBI uptake for detoxification but also may play a role within the reduction of metabolic stress through regeneration of intracellular NAD(P)H/NAD(P)^+^ levels. Furthermore, we illuminate the thermodynamics that governs iron transport, namely the redox potential cascade and electrochemical behavior of key components of the electron transport systems that facilitate the movement of electrons across the plasma membrane to the extracellular compartment. We also take account of kinetic changes that occur to transport iron into the cell, namely membrane dipole change and their consequent effects within membrane structure that act to facilitate transport of ions.

## The role of iron and its uptake into mammalian cells

Iron is an essential micronutrient required for normal functioning in mammals (Lieu et al., 2001; Pantopoulos et al., 2012). Iron's role as a key element revolves around its abundance in nature and its multiple oxidation states (being a d-block transition metal), which allow both electron transfer and the binding to many different ligands. This combination of attributes allows adaptation and control of iron's chemical properties to suit the requirements of the cell and results in its essential role in many processes (Hayashi and Stuchebrukhov, 2010; Kamga et al., 2012).

This review initially provides a background of the overall iron transport in the whole organism, to contextualize later in depth discussion about the specific mechanisms within iron uptake at the cell level.

**Figure 1:**
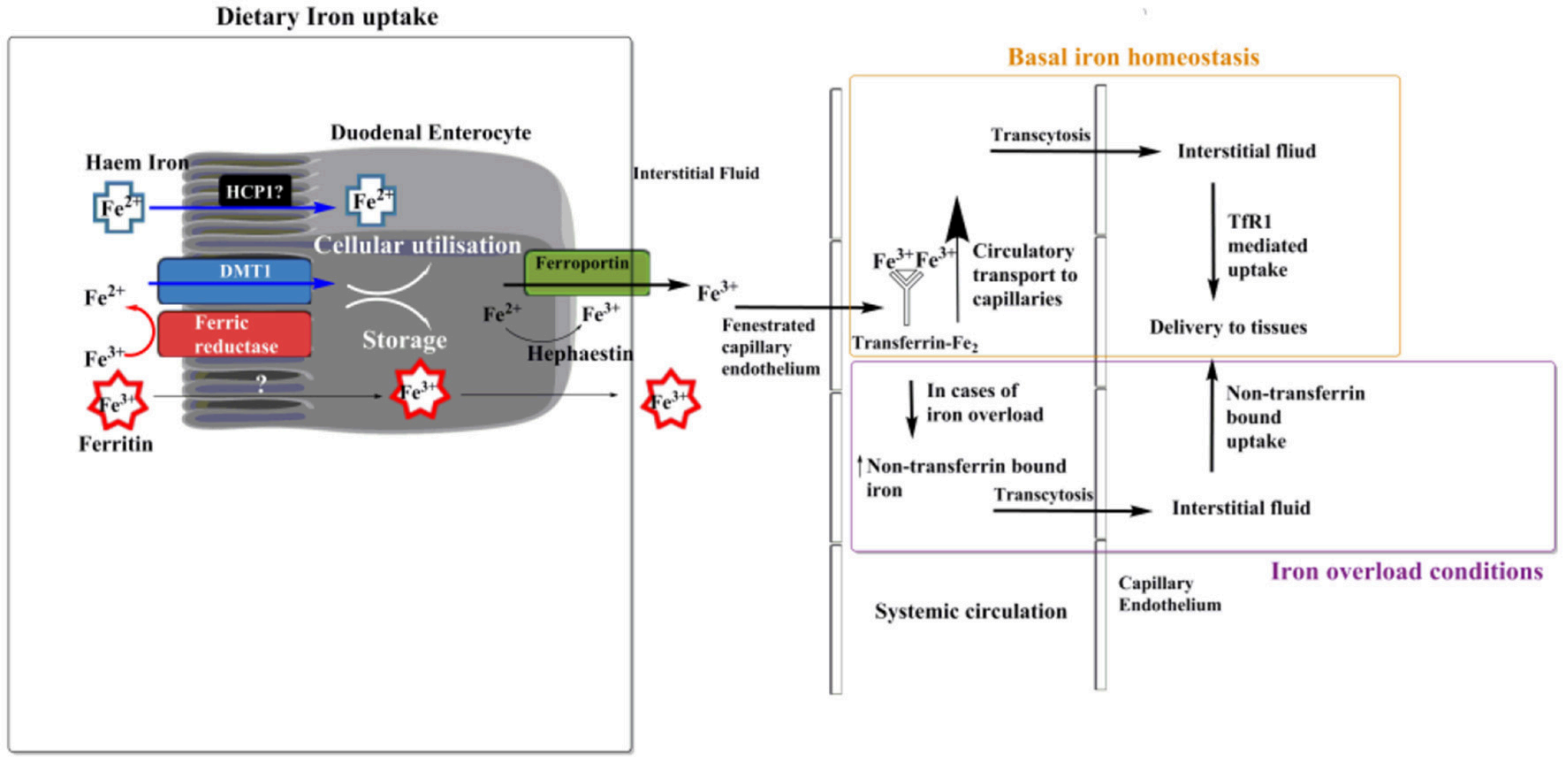
Transport of dietary iron. Ferrous iron can enter the enterocyte cell in the form of haem. The exact method of haem uptake is yet to be elucidated, but Haem Carrier Protein 1 (HCP1) has been suggested to fill this role. Non-haem ferrous iron can enter the enterocyte cell in the form of ferritin via receptor mediated endocytosis, although a specific receptor is yet to be identified (Theil, 2004; Kalgaonkar and Lönnerdal, 2009). The main route of iron uptake in the duodenum is of “free” ferric iron, via a transferrin-independent mechanism. Iron is first reduced into a soluble form via a ferric reductase enzyme at the brush border of duodenal enterocytes. Transport into the enterocyte is then facilitated by the divalent metal transport 1 (DMT1). Once inside the enterocyte cell ferrous iron can be utilized for intracellular processes such as haem and Fe-S cluster biosynthesis, or stored in the molecule ferritin. Export primarily occurs, which is facilitated by ferroportin, at this stage iron is oxidized back to its ferric form by hephaestin. Most ferric iron passes through the fenestrated capillary endothelium of the intestine before being bound by the glycoprotein transferrin, where it is circulated within the plasma. Here transferrin bound iron can pass via transcytosis (Williams et al., 1984) across the capillary endothelium and into the interstitial fluid where it is delivered to tissues via transferrin receptor 1 (TfR1) mediated uptake (under normal physiological conditions). In cases of iron overload a pool of non-transferrin bound iron (NTBI) is formed, in which case iron uptake into most mammalian cells can also occur via NTBI uptake (Brissot et al., 2012).

Dietary iron can come in two forms: haem bound or non-haem bound. Haems are a class of porphyrin that contain ferrous iron. Uptake of haem bound iron may occur across the duodenal brush border, although the exact mechanism is not fully elucidated (Sharp and Srai, 2007). Haem carrier protein 1 (HCP1) has been suggested as a potential uptake transporter for haem (Shayeghi et al., 2005), but recently it has been proposed that HCP1 may be a folate transporter unrelated to haem (Qiu et al., 2006). Non-haem uptake is believed to occur *via* two methods. A less studied, less used method is ferritin uptake by enterocytes (Kalgaonkar and Lönnerdal, 2009). Theil (2004) suggested that food-derived ferritin could be used as another source of dietary iron, and Kalgoenkar et al. showed that in Caco-2 intestinal cells ferritin uptake occurs via receptor mediated endocytosis, but the receptor which binds ferritin and is then internalized is yet to be identified. It is important here to note that iron is stored within ferritin in its ferric form, Fe^3+^ (Figure 1). They also state that at high ferritin concentration transport may occur via micropinocytosis. The second pathway of the transferrin mediated uptake of non-haem iron is thought not to occur at the brush border of duodenal entercoytes (Srai et al., 2002), but may occur elsewhere within the gastrointestinal tract. In this context, some studies have reported that apotransferrin is not present within the duodenum of rats (Idzerda et al., 1986) or humans (Pietrangelo et al., 1992). In addition, and although transferrin receptors are found in the villous epithelium (Banerjee et al., 1986) and mucosal cells of duodenum (Huebers et al., 1983), they are not found at the brush border (Parmley et al., 1985; Banerjee et al., 1986). These raise the question of how pivotal transferrin's role is for iron internalization by duodenal enterocytes and its subsequent trafficking. It has been suggested that a predominant method of non-haem iron uptake by duodenal enterocytes at the brush border is “free” ferrous iron uptake, facilitated by the Divalent Metal Transport 1 (DMT1) (Canonne-Hergaux et al., 1999). However before cellular internalization can occur via these transporters, ferric (Fe^3+^) iron must be reduced to ferrous (Fe^2+^) form, which can be achieved by an enzyme capable of ferrireductase activity. There is a number of enzymes that can catalyze this reduction step, but the predominant candidate within the duodenum is duodenal cytochrome b (Dcytb) (Latunde-Dada et al., 2002).

Following transport across the apical cytoplasmic membrane of enterocytes into the cytoplasm by one of the above suggested mechanisms, ferrous iron can be utilized or sequestered within the cell, or exported in apical-basolateral direction across the cell. These former processes commonly occur through biosynthesis of Fe-S clusters of the electron transport chain (Hayashi and Stuchebrukhov, 2010) or storage in the form of ferritin (Arosio et al., 2009), respectively. Storage in mammalian ferritin is facilitated by ferrous iron oxidation, and subsequent storage, in addition to detoxification via the consumption of toxic peroxides involved in the Fenton reaction (Arosio et al., 2009). The 480 kDa, 24-subunit ferritin protein a spherical, but hollow, structure and can accommodate up to 4,500 ferric iron atoms at any one time (Finazzi and Arosio, 2014). However it represents a very small amount of circulating iron and is more abundant within the cell than in the circulatory system (Arosio et al., 2009). Ebrahimi et al. have proposed a hypothesis for the ferroxidase capacity of ferritin that facilitates iron storage as Fe^3+^ (Honarmand Ebrahimi et al., 2012). Ferritin can exert ferroxidase activity through its active center and they suggested that this center consists of two binding sites that can both bind ferric and ferrous iron, but with different affinities. There is, in addition, a gateway to the center present in the form of a third binding site. Through the binding and catalytic oxidation of two ferrous iron ions, a ferric iron loaded center, Fe^3+^-O(H)–Fe^3+^, is generated and forms a stable product. When a new ferrous ion binds, the existing ferric iron is displaced and the new ion undergoes oxidation to produce ferric iron thus forming the next stable intermediate. The displaced ferric iron is presumed to form the core of the ferritin as the method of iron storage, and is stored as ferrihydrite (Norberg and Rayner, 1987; Chasteen and Harrison, 1999). A more recent review paper also published by Ebrahimi et al. (2015) provides more insight into the chemistry of the catalytic center of ferritin and builds upon their previous work. Each sub-unit of ferritin that is involved in the process of ferrous iron oxidation possesses three metal-ion binding sites, as previously hypothesized. Within eukaryotes, two of these sites form a di-nuclear metal-ion binding site, named the ferroxidase center, whilst the third forms an additional metal-ion binding site close to the ferro-oxidase center.

If not stored or utilized within the enterocyte then ferrous iron is exported into the circulatory system. Possible mechanisms of the export across the basolateral cytoplasmic membrane of the enterocyte include involvement of ferroportin (Dunn et al., 2007) (the only current putative iron exporter identified to be involved within iron export throughout the whole body) which works in concert with the ferroxidase hephaestin (a gut homolog to the abundant plasma protein ceruloplasmin) to oxidize iron to its ferric form during the export (Vulpe et al., 1999; Anderson and Frazer, 2017; Bonaccorsi di Patti et al., 2018). Such exported ferric ions cross the mucosal interstitial fluid and pass through the fenestrated capillary endothelium into the bloodstream, whereby complexation by transferrin occurs (Morgan and Oates, 2002). This was demonstrated by Morgan et al. (Morgan and Oates, 2002) by measuring iron levels in the lymph and plasma of rats after administration of Fe^3+^. They found that only 4% of iron was present in the lymph, whilst the remainder entered directly into the blood. They state, “If transferrin had acted as the carrier of iron from the absorptive cells most of the iron should have been transported by the lymph because retrieval of extravascular protein, including transferrin, to the circulation occurs mainly by this route.” Therefore it is stated that transport of iron into the intestinal bloodstream occurs in its molecular form.

Transferrin (Tf) is an 80-kDa glycoprotein mainly synthesized by hepatocytes (Morgan, 1981). It is the major molecule to complex ferric iron (Fe^3+^) in the plasma and extracellular fluids, and is used to transport iron throughout the circulatory system in its ferric form (Fe^3+^). Having been exported from enterocytes and entered the circulation, ferric iron is complexed by transferrin. Iron bound to transferrin can then be transported throughout the body via the circulatory system. Transport in the blood plasma, allows the iron-transferrin complex to transcytose across the capillary endothelium (Williams et al., 1984) and deliver transferrin bound iron to interstitial fluid of different tissues in the body. Transcytosis involves the receptor mediated endocytosis of the transferrin-iron complex, within vesicles that subsequently traverse the cell and are exported via exocytosis on the luminal side (Tuma and Hubbard, 2003).

Transferrin has an extremely high affinity for ferric iron (Aisen, 1978) at 10^24^ M^−1^ and, under normal conditions, only 30% of the body's transferrin is saturated with ferric iron (Anderson, 1999). Due to this, it is thought that nearly all ferric iron in the systemic circulation and interstitial space is in a transferrin bound form under normal conditions. In a healthy individual non-transferrin bound iron (NTBI) in the plasma may still exist, but it does not exceed 1 μmol/L and, is commonly undetectable (Anderson, 1999). This however will differ in cases of iron overload (Fleming and Ponka, 2012), where saturation of transferrin occurs resulting in an increase in free, circulating ferric plasma iron, not bound to transferrin. This free iron is referred to as NTBI (Hershko and Peto, 1987). (The term NTBI generally refers to all iron not bound to transferrin, but is more commonly used to refer to a low molecular weight pool of iron within the plasma) (Breuer et al., 2000; Hentze et al., 2004). Certain diseases, such as hereditary hemochromatosis, can cause an increase in circulating NTBI (Allen et al., 2008). Conversely, there are instances where iron deficiency can occur, generally from blood loss or poor dietary absorption, such as in celiac disease (Goddard et al., 2011). In cases where the amount of NTBI increases cells use a non-transferrin mediated method of iron uptake, similar to that of duodenal uptake at the brush border (Lane et al., 2015).

Most human tissues express internalizing transferrin receptors (at least at low levels) (Daniels et al., 2012), such as the transferrin receptor 1 (TfR1), and so following transport from the systemic circulation into the interstitial fluid transferrin-mediated uptake facilitates the internalization of the Tf-Fe^3+^ complex from the interstitial fluid into cells.

## Two pathways for iron uptake: different roles for iron homeostasis

It is clear that there are two main methods of cellular iron uptake. The first is the more basic transferrin-independent system, which is predominantly used when damaging concentrations of NTBI are present. The second is the transferrin-dependent pathway, which is used to meet the need for strictly controlled iron homeostasis. Evidence for this is observed when looking at how the regulation of the transferrin dependent and independent pathways differs by the cell (De Silva et al., 1996). The transferrin receptors are upregulated when intracellular iron levels are low (iron deprivation), but there is no upregulation of the non-transferrin bound pathway (Kaplan et al., 1991; Inman and Wessling-resnick, 1993). Conversely, we see upregulation of the non-transferrin pathway when systemic iron overload within the circulation occurs, in a number of cell types (De Silva et al., 1996). This leads to the literature's consensus that the transferrin-mediated system has developed out of a need for strict internal control of iron whereas non-transferrin uptake is employed within crisis, such as iron overload or in cases where less tightly controlled iron uptake is required, such as for dietary absorption. Interestingly, besides the gut there is another area of the body where NTBI uptake systems are routinely employed—the lung. The lung epithelium is regularly exposed to environmental iron as a consequence of its function. The lung epithelium therefore uses NTBI uptake as a mechanism to sequester and detoxify iron within the cell (Ghio et al., 2006; Ghio, 2009). This lends support to the consensus that NTBI uptake occurs during the diseased state as a crisis mechanism during iron overload.

There is evidence to suggest the process of NTBI transport may have multifaceted functionality, by utilizing the transplasma electron transport system (tPMETS) that is used to reduce extracellular iron. One function is that cells may use this pathway to detoxify iron when iron overload (De Silva et al., 1996) occurs, through the reduction, uptake and sequestration of ferric iron. Another is use in times of metabolic stress. Evidence for the latter was given by studying iron reducing capacity of respiratory competent vs. incompetent Namalwa cells (Larm et al., 1995). It was observed that respiratory incompetent cells reduced ferricyanide at three times the rate of respiratory competent ^+^Namalwa cells (Larm et al., 1995). This is because aerobic respiration was absent and due to this, the cell needs to run the less efficient glycolytic pathway at a greater rate to provide sufficient energy. Regeneration of NAD^+^ from NADH is achieved by the transfer of electrons from NADH to an external electron acceptor by the tPMETS. This regeneration is reflected in the greater rate of reduction of extracellular ferricyanide. These findings are important as they support the hypothesis that the tPMETSs that facilitate NTBI uptake actually have multifaceted functionality, either being used to protect the cells in times of stress or to adsorb iron from the gut. Interestingly, this NADH/NAD^+^ equilibrating function appears to also occur within other cell types when mitochondrial activity is removed, albeit in relation to tPMETSs involved in cell surface oxygen consumption. Herst et al. (2004) demonstrated that HL60p^0^ cells, which are devoid of mitochondrial DNA, upregulated surface tPMETSs facilitate the regeneration of NADH/NAD^+^ levels to allow for aerobic glycolysis to occur. For this reason, tPMETS are thought to benefit rapidly propagating cells by yielding a metabolic advantage through the regeneration of NAD(P)^+^ at increased rates. Thus, increased numbers of tPMET centers supports higher flux through glycolysis, for example in anoxic conditions.

Where the literature is vague in respect to iron transport is not the macro-level transport through the body compartments, or even the methods of uptake, but in the underlying biophysical and electrochemical mechanisms which underpin the processes occurring. There is much contention in the literature relating to the actual mechanisms which are occurring during tPMET, especially within NTBI uptake. As such, we have sought to the review contemporary literature regarding the non-transferrin bound pathway, in addition to summarizing literature on the electrochemical control of the tPMET that provides the foundation for “free” iron transport. By combining this with our highlighting of the multifunctionaility of non-transferrin bound uptake systems, and specifically the tPMET component of these, this review succinctly and extensively provides new insights into how NTBI is both utilized and transported by the body.

### The transferrin-bound pathway

The method by which iron is transported depends on whether the iron is bound to transferrin, or not. This section will describe the transferrin-bound pathway in detail.

**Figure 2:**
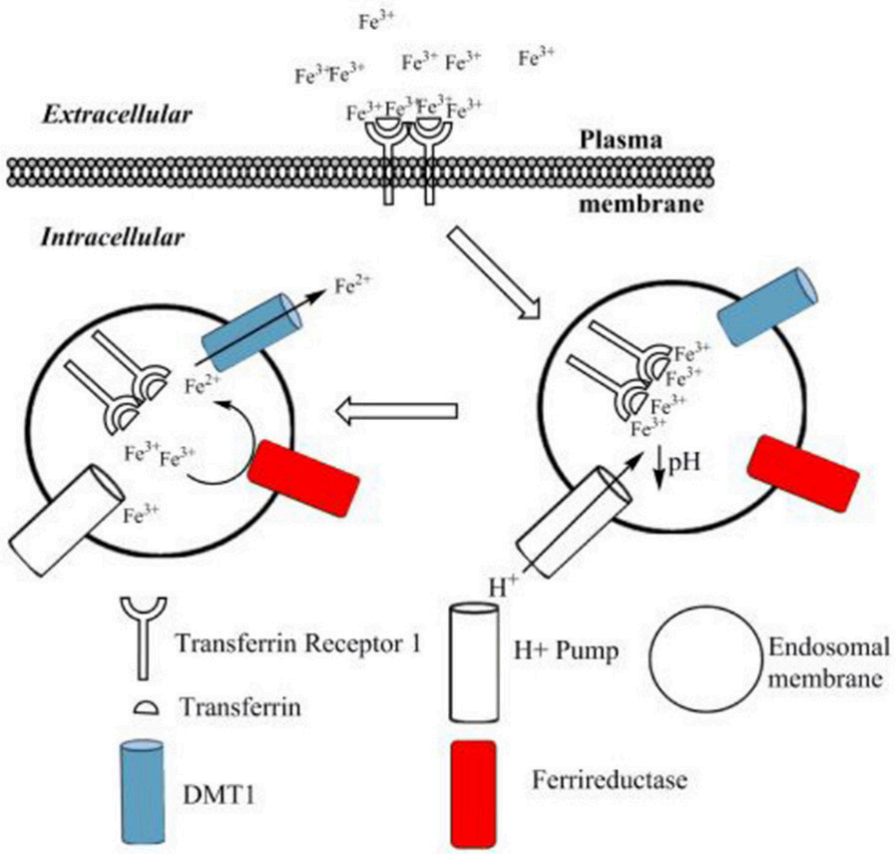
The transferrin bound cycle of iron uptake. Ferric iron binds to transferrin to form holo-transferrin (Tf). This di-ferric complex then binds to the transferrin receptor 1 (TfR1). Upon binding to the TfR1, the whole complex is internalized via receptor-mediated endocytosis. An ATP driven H^+^ pump drives a pH reduction within the endosome, which causes dissociation of ferric iron from transferrin. The unbound ferric iron is reduced via a transmembrane ferri-reductase to form ferrous iron. The ferrous iron is then transported into the cytoplasm by the DMT1 transporter. The receptor and transferrin protein are then recycled to the surface, ready for the cycle to repeat (not shown). Adapted from Lawen and Lane (2013).

Transferrin (Tf)-bound iron cellular uptake (Figure 2) is a well-understood mechanism of transmembrane iron transport into cells, which is extensively described within the literature (Dautry-Varsat et al., 1983; Ohgami et al., 2005; Lane and Lawen, 2008; Lawen and Lane, 2013). Transferrin binds with high affinity to iron in its ferric form, as discussed. A transferrin-ferric iron complex (Tf-Fe) can exist in several forms: apo-Tf, monoferric-Tf or diferric-Tf (holo-Tf) (Lawen and Lane, 2013); the monomeric form in turn has two possible variants, depending on iron's preference for either the N or the C lobe of transferrin (Makey and Seal, 1976). This binding depends mostly upon the environmental pH, with the maximal binding occurring at pH 7.4 (Aisen and Listowsky, 1980; Cheng et al., 2004). Normal conditions usually result in a transferrin saturation of roughly 30% (De Domenico et al., 2008; Pantopoulos et al., 2012). The Tf-Fe uptake is mediated via the transferrin-receptor-1 (TfR1). This 95-kDa transmembrane receptor is a homodimer which preferentially binds holo-Tf (Aisen, 2004). TfR1 is expressed in most cells, with only a few exceptions, for example mature erythrocytes (Enns et al., 1982). Upon binding of holo- or monomeric-Tf to the TfR1, the entire complex is internalized via receptor-mediated endocytosis, involving clathrin-coated pits (Qian et al., 2002). Within 2–6 min (Dautry-Varsat et al., 1983) the internalized endosome experiences a decrease of pH to 5.3–5.6 (Dautry-Varsat et al., 1983; Klausner et al., 1983). This change in pH is driven by a vacuolar-type H^+^-ATPase (V-ATPase), a transmembrane protein of the endosome (Presley et al., 1997). The resulting acidic environment causes ferric iron to dissociate from Tf (Dautry-Varsat et al., 1983; Paterson et al., 1984), whilst the now apo-Tf remains bound to the TfR1. Recent work by Eckenroth et al. (2011) have shown the binding of Tf to its receptor induces structural changes within the TfR ectodomain that prime Tf for release, this occurs through alteration of the Tf-TfR1 interaction upon a change to acidic pH.

Following release from the transferrin molecule, the dissociated ferric iron is reduced via ferri-reductases. It is important to note this process occurs on the endosomal membrane, and there are both similarities and differences in relation to the non-transferrin bound pathway. In this transferrin-bound pathway, the ferri-reductases used to achieve this are the 6-transmembrane epithelial antigen of the prostate-3, steap3 (Ohgami et al., 2005), and/or members of the cytochrome b_561_ family (Lane and Lawen, 2009). Once ferrous iron has been formed, transport out of the endosome is facilitated via DMT1, as discussed previously (Andrews, 1999; Garrick et al., 2003). It has also been suggested that ZRT/IRT-like protein (Huebers et al., 1983) (ZIP14) can facilitate ferrous iron transport out of the endosome (Tripathi et al., 2015). Ferric iron reduction occurs because the DMT1 transporter only transports iron in its ferrous form, in addition to iron being required in its soluble, reduced form. The DMT1 and cytochrome b_561_ family are also used in the non-transferrin pathway, but steap3 is not, and is only used at the endosomal membrane of the transferrin-bound path. Finally the apo-Tf/TfR1 complex is recycled back to the cell membrane to be re-used (Morgan, 1996). It is here, at physiological pH, that apo-Tf dissociates from the receptor (Bomford et al., 1985). In this way the transmembrane ferri-reductase-DMT1 system present in the endosomal membrane is used to facilitate ferric iron movement into the intracellular compartment.

### The non-transferrin bound pathway

The second method of non-haem iron uptake into mammalian cells is that of a non-transferrin bound pathway. This pathway uses transplasma membrane electron transport (tPMET) at the cell plasma membrane to reduce iron, as opposed to the endosomal membrane tMET that occurs within the transferrin-bound pathway. The presence of multiple iron uptake systems, which the cell can exploit, are predicted to have arisen from how evolutionary biology has steered cellular iron homeostasis. Most prokaryotes and single-celled eukaryotes, like multi-cellular organisms, require iron. Due to the high amounts of iron that is in inaccessible forms these organisms have had to develop various methods to acquire the environmental iron they may encounter (Wandersman and Delepelaire, 2004). Siderophores (Neilands, 1995), which are low molecular weight ferric iron chelators, are one way in which bacteria seek to harvest iron from their surroundings. Other mechanisms include acquisition of iron from host systems (Guerinot, 1994), or the use of ferri-reductases to reduce iron prior to transport across the plasma membrane (Guerinot, 1994; Shatwell et al., 1996). Much like the latter method for acquisition within single-celled organisms, it is thought that most eukaryotes possess a system of ferric iron reduction and subsequent transport that can be used to obtain iron from their surroundings in the event of iron overload and the risk of iron toxicity, except for the absorptive intestinal cells that acquire iron for dietary needs, and for iron detoxification at the lung-air interface. The following section outlines the tPMETSs involved in the non-transferrin bound uptake pathway.

## Enzyme-mediated electron transfer: a general model

**Figure 3:**
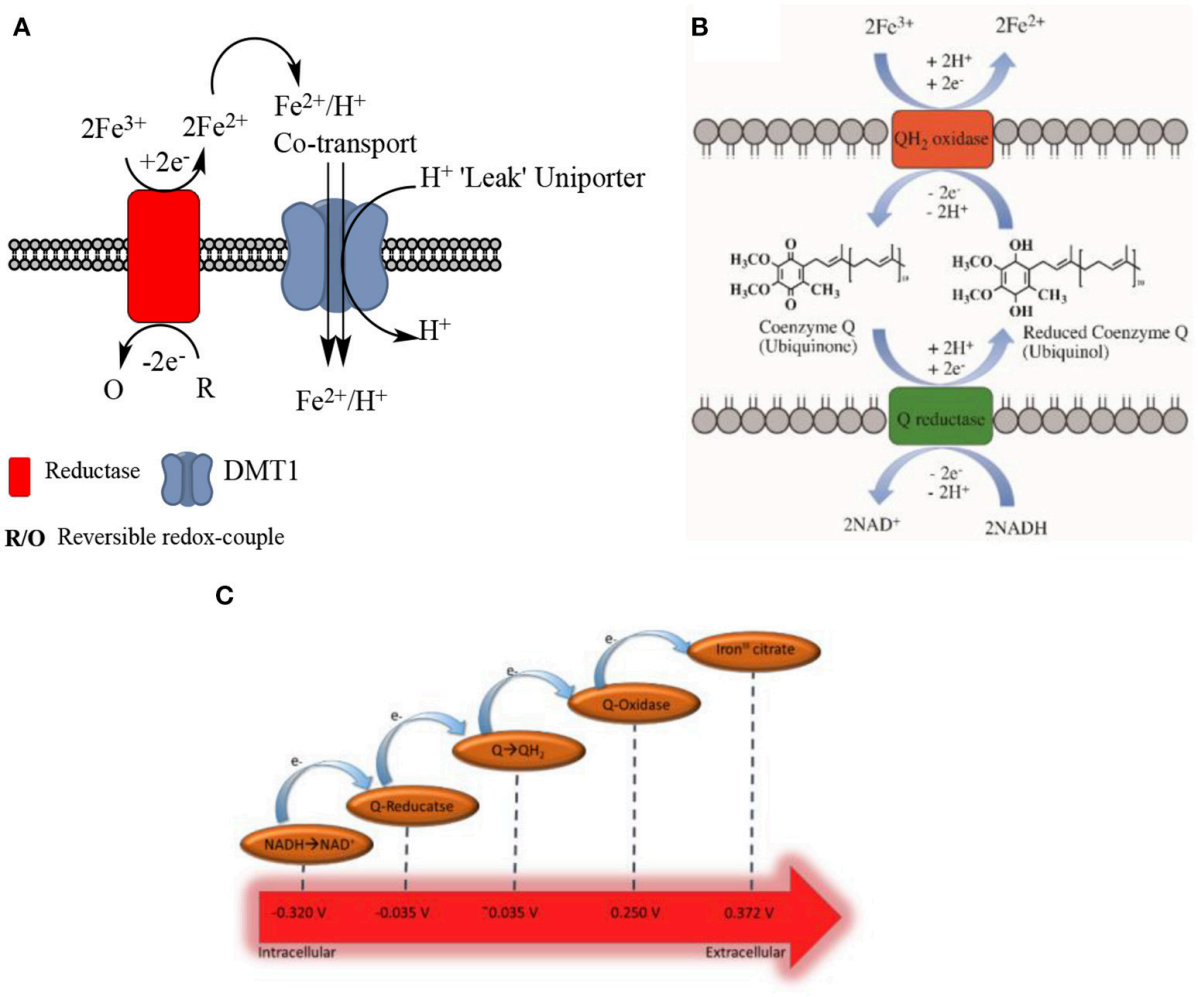
**(A)**. Generalized, traditional model of non-transferrin bound iron uptake. A reversible redox couple provides electrons to the ferri-reductase, through its oxidation from a reduced to an oxidized form. Facilitated by the reductase, these electrons are used to reduce two molecules of ferric ion to form ferrous ion in the extracellular compartment. This ferrous ion is co-transported across the cell membrane with H^+^ ions, into the intracellular space; this is facilitated by the DMT1 transporter. The DMT1 transporter also acts as a uniporter, and “leaks” H^+^ ions to the intracellular compartment from the extracellular compartment**. (B)** Ubiquinone shuttle of NADH-ferri-reductases. It has been shown that coenzyme Q can facilitate electron transport through the membrane of cells for the NADH-ferri-reductases (Oakhill et al., 2008). This schematic proposes a method of how this can occur, based upon the mechanisms of coenzyme Q electron shuttling within complex I and II of the electron transport chain. **(C)** Schematic of the favored electron transfer pathway in a general transplasma membrane electron transport system according to the (in some cases approximated) standard redox potential at pH 7.

Biological membranes exist to enable cells and their organelles to control their environment by acting as selectively permeable barriers. Redox centers exist in membranes that are involved in bioenergetics and are located both in cytosolic structures such as in the endoplasmic reticulum, and in the plasma membrane. These redox centers function to transport electrons across membranes and are known to be involved in iron reduction. The centers have been termed as either (trans)plasma membrane electron transport systems (tPMETS or PMETS) or plasma membrane oxido-reductase (PMOR) centers (Berridge and Tan, 1998, 2000; Baker and Lawen, 2000). A general model for tPMETSs that involves naturally occurring electrochemical mediators has been proposed (Figures 3A,B) (Morré and Morré, 2004). This general model consists of a redox enzyme system that spans the plasma membrane and is comprised of three components. One component faces the cytoplasm, another element acts within the hydrophobic lipid bilayer and the third interfaces with the cell's external environment. The ferri-reductase used as a tPMETS, as well as the redox couple employed, can differ; this will depend on where the system is being employed. Interestingly, evidence has already been reported that there is more than one membrane redox system capable of reducing ferric iron (Rawson et al., 2014) in eukaryotes.

**Figure 4:**
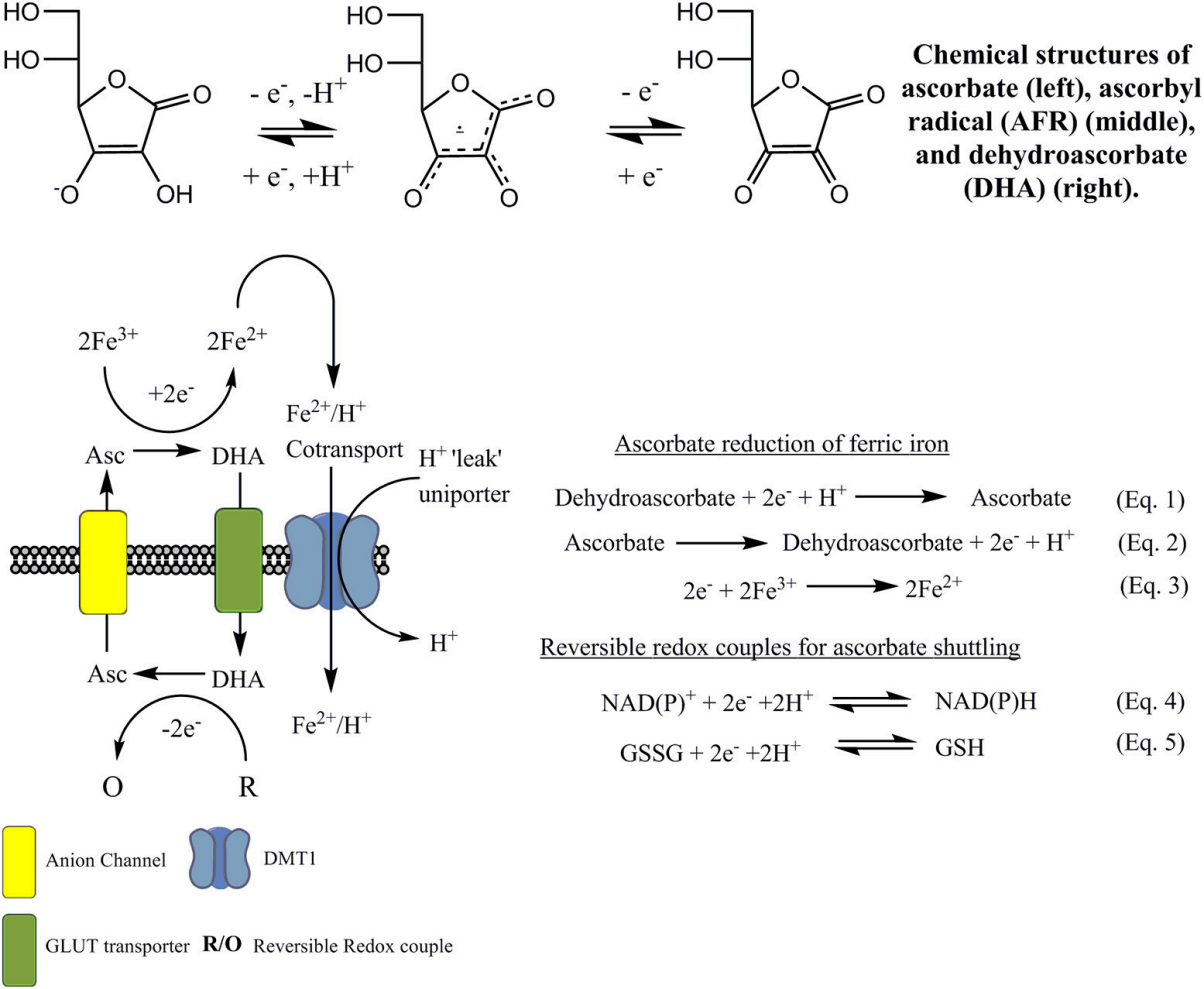
Current ascorbate cycling model for NTBI reduction and uptake. Ascorbate provides the necessary reducing equivalents to reduce ferric iron to ferrous iron, by becoming oxidized to DHA. Fe^2+^ is then transported, putatively by DMT1, across the membrane. DHA is now cycled back to the intracellular phase via glucose transporters such as GLUT1. Internal regeneration of DHA to ascorbate occurs via direct two-electron reduction using glutathione/glutathione disulfide couple (GSH/GSSG). Reduction also occurs enzymatically using NADPH and GSH dependent enzymes. The regenerated ascorbate crosses the membrane via anion channels and the process repeats.

Ferri-reductase systems have been extensively covered in the literature. There are several of these redox systems that have been proposed, and it has been suggested they are of “two types” (Lane and Lawen, 2009). One is dependent on enzyme-mediated iron reduction while the other relies on a natural redox mediator, such as ascorbate, and is independent of enzyme-iron interactions which will be discussed later in the text (Figure 4). The intracellular facing part of the general enzymatic based tPMETS (Figures 3A,B) functions by undergoing redox reactions, utilizing intracellular redox mediators. These are known to occur with the nicotinamide adenine dinucleotide phosphate couple (NAD(P)H/NAD(P)^+^) (Baker and Lawen, 2000) (Figure 4, Equation 4), ascorbate/dehydroascorbate (Asc/DHA) couple (Figure 4, Equations 1, 2) (Van Duijn et al., 1998), and suspected to occur with the glutathione/glutathione disulphide couple (GSH/GSSG) (Figure 4, Equation 5). The lipophilic component of the tPMETS (Figure 3B), that mediates electron transfer to the outward facing component, is proposed to be Coenzyme Q. Coenzyme Q is synthesized in mammals by all cells (Navas et al., 2007), and is present within all membranes of the cell (Kalén et al., 1987; Sun et al., 1992). Coenzyme Q's role has been well-documented in transporting NADH derived electrons through the membrane, from one complex of the Electron Transport Chain to the next (Lodish et al., 2007). In addition, it has been demonstrated that for NADH oxido-reductases, the hydrophobic coenzyme Q, also known as ubiquinone, is responsible for the shuttling of electrons to the ferri-reductase (Sun et al., 1992).

In the case of the NADH dependent ferri-reductase, which is an enzyme based tPMET (Figure 3A), oxidation of intracellular NADH to NAD^+^ causes the production of free electrons and a H^+^ ion. The electrons and the H^+^ ion, and an additional intracellular H^+^, then cause the reduction of coenzyme Q to form reduced coenzyme Q, or ubiquinol (with the addition of 2 H^+^). The subsequent reduced coenzyme Q molecule shuttles the electrons through the membrane to be used to reduce extracellular ferric iron. It is important to note that the use of reduced coenzyme Q for shuttling is localized in the membrane.

### Enzyme based ferrireduction: the enzymes that fit the classic model

A number of different ferri-reductases have been identified, which fit the classical model discussed above (enzyme-mediated electron transfer), however they differ both in structure and in the site where they are expressed. tPMETSs have been shown to be multicomponent (Lesuisse et al., 1996). The proteins of each component have been segregated from cellular membranes and their sequence has been elucidated. It has been demonstrated that the sequences of the tPMET components are closely related to cytochrome b5, cytochrome c, protein disulphides and various ferrichelates (Morré and Morré, 2004). The redox half-wave potentials of, for example, cytochrome b5 and cytochrome c, that display similar sequence homology to isolated tPMETS components, are known to span a wide range from approximately between −0.035 (Aono et al., 2010) (for the reductase) to 0.25 V (Eddowes et al., 1980) (for the oxidase) vs. Normal Hydrogen Electrode. Importantly, from a thermodynamic view, the potential of the reductase is significantly more positive than that of the NADH/NAD^+^ (−0.324 V) or NADPH/NADP^+^ (−0.339 V) couples, while the potential of the oxidase is more negative than that of the Fe(II)/(III) couple which results in electron transfer via a Q-reductase. As shown in Figure 3C, this allows for electron flow from the intracellular to the extracellular compartment. It is thought this is to increase the solubility of the iron and thereby facilitating intracellular iron transport (more detail is given in later sections). However, much work is still to be performed to elucidate the exact structure and function of tPMET centers, and to provide a more complete understanding of their roles in the cell.

It is currently hypothesized that in enterocytes duodenal cytochrome-b ferri-reductase (Dcytb) reduces ferric ion at the apical membrane in preparation for iron uptake. However, some studies have shown that the level of iron uptake does not significantly reduce in mice with Dcytb-knockout (Gunshin et al., 2005). Dcytb is highly expressed at the duodenal enterocyte brush border and is a member of the cytochrome b_561_ family (Oakhill et al., 2008). The cytochrome b561 family displays strong sequence homology between its members, and constitutes a group of multi-pass transmembrane enzymes found only in eukaryotes (Lu et al., 2014). Dcytb is a 286 amino acid, six transmembrane domain protein (Wyman et al., 2008), that acts as an oxidoreductase that transfers electrons from cytosolic ascorbate to extracellular electron receptors (Vlachodimitropoulou et al., 2010). It has been shown to be expressed in a wide variety of cell types including human erythrocytes (Su et al., 2006), lung epithelium (Turi et al., 2006), K562 cells (Kovar et al., 2006), astrocytes (Loke et al., 2013), Caco-2 (Balusikova et al., 2009), and HEP-G2 cells (Anderson, 1999). Four of its transmembrane helices contain a single histidine haem-binding residue, which is organized to construct two iron coordination complexes. Development of this his-binding model resulted in identification of two potential substrate-binding sequences, which are conserved between the cytochrome b_561_ family (Okuyama et al., 1998). The first sequence, ALLVYRV, between residues 68 and 74, is located at the end of alpha-helix II. It forms an ascorbate recognition/binding region with Lys83. The second, SLHSW is between residues 119 and 123 and is thought to adopt a location between helices II and III. This sequence is proposed to form another ascorbate or DHA-binding site (Okuyama et al., 1998). This sequence binding data supports the enzyme-mediated explanation where ferric iron is reduced by intracellular electron donors such as ascorbate. The most current characterization of the Dcytb protein by Ganasen et al. (2018) suggests an electron transfer pathway whereby an electron from intracellular ascorbate passes to extracellular iron or the ascorbyl free radical via two histidine-coordinated hemes.

Another ferri-reductase, previously mentioned as part of the transferrin-dependent pathway, is Steap3. This STEAP family protein is partially homologous to the oxidoreductases found in bacteria and archaea, and to the FRE metalloreductase in *Saccharomyces cerevisiae* (Ohgami et al., 2005, 2006). It plays a key role in the reduction of ferric iron within the endosome of erythroid cells, following the internalization of the Tf-ferric iron complex via endocytosis. It is this reduction that occurs prior to transport of the reduced ferrous iron into the intracellular compartment. Steap3 is comprised of a C-terminal transmembrane domain, which is thought to coordinate a single intramembrane haem. An N-terminal cytosolic oxidoreductase domain is also present which uses cytosolic NADPH as a cofactor and is predicted to also use flavin as cofactor to reduce ferric iron because of the presence of a putative flavin binding site (Ohgami et al., 2005; Sendamarai et al., 2008).

The electron transfer process is proposed to consist of sequential electron transfer from cytosolic NADPH to the flavin and intramembrane haem, and finally to the ferric iron within the endosome (Sendamarai et al., 2008). The mechanism of electron transport, mediated by these ferri-reductases, is both relevant and important to further understanding the intricacies of these systems. It has been suggested that the electron transport mechanism that Steap3 exhibits is a gated system, where electron flow into the endosome only occurs in response to a need for ferric iron reduction. An obvious possibility for how this selective mechanism works is a structural change in response to ferric iron interaction, thus allowing electron flow into the endosome. The structural clarification of the oxidoreductase domain by Sendamarai et al. points to other possibilities (Sendamarai et al., 2008). Due to the proximity of the active site to the dimer interface of Steap3, the oligomeric state of the enzyme may strongly affect the catalytic ability of the enzyme and regulate its oxidoreductase activity (Sendamarai et al., 2008). Another possibility is that Steap3 is mediated by other members of the transferrin-bound pathways, such as DMT1.

### Enzymes of the non-classical model

Although tPMET centers described above have been postulated, other centers with variations on this theme have been proposed to exist, such as purely cell-surface based complexes (Berridge and Tan, 1998). Lawen et al. (2005) have shown that the mitochondrial voltage-dependent anion-selective channel 1(VDAC1) that also exists in the cell membrane, acts as a ferricyanide reductase at that site (De Pinto et al., 2003). Voltage-dependent anion-selective channel 1 (VDAC1) is a 30–35 kDa protein, which is localized within the outer mitochondrial membrane. The function of VDAC1 within the plasma membrane was unknown, until Baker et al. published data showing that VDAC1 was in fact a NADH-ferricyanide reductase (Baker et al., 2004). They used immunoprecipitation directed against VDAC1 to show that preparations purified from plasma membrane and mitochondrial fractions that exhibited NADH ferricyanide reductase activity were in fact VDAC1. An additional publication by Baker et al. succinctly reviews the data for NADH-oxidoreductases, and may prove useful for readers of this review (Baker and Lawen, 2000). It is likely that more membrane sites capable of iron reduction will be identified (Rawson et al., 2014).

### The ascorbate shuttle model

Another non-classical iron reducing transporter system that has been described involves the membrane electron mediator ascorbate. Ascorbate, more commonly known as vitamin C, is maintained in the cell at an outward facing concentration gradient (high intracellular concentration). This gradient is generated and maintained mostly via the sodium-dependent import of ascorbate into cells by sodium-ascorbate co-transporters (SVCTs 1 and 2) (Savini et al., 2008). In addition to the SVCTs, the mammalian facilitative glucose transporters, GLUT1, GLUT3, and GLUT4, can transport dehydroascorbate (DHA) into the cell, where it is then rapidly reduced to ascorbate, thus maintaining both the DHA and ascorbate gradients. Ascorbate is also released from the cell via anion channel transport, but the physiological functions of this are not entirely clear.

One of the first suggestions of a mechanism that uses intracellular ascorbate accumulation as opposed to an NADH-dependent ferrireductase was from Van Duijn et al. (1998). In this paper they identified that loading HL-60 myeloid leukemic cells with ascorbate stimulated ferricyanide reductase activity in a mechanism distinct from the NADH-ferricyanide reductase pathway. However, they did not elucidate whether this was due to a shuttling mechanism or, as we now know, the potential utilization of intracellular ascorbate by enzymes such as Dcytb.

Lane et al. previously suggested that DHA can undergo reduction by an electron shuttle mechanism to form ascorbate (Figure 4, Equation 1), which crosses the plasma membrane via transmembrane anion channels and through its own oxidation (Figure 4, Equation 2) acts directly to reduce extracellular ferric iron (Lane and Lawen, 2008, 2009) (Figure 4, Equation 3). Indeed, this mechanism has recently been shown to be active in plants (Grillet et al., 2014). This process is thermodynamically favorable as ascorbate-DHA couple has a standard redox potential of +0.058 V (pH 7) vs. the 0.372 V (pH 7) of a typical iron chelate, such as iron^III^ citrate (Figure 3C).

Intracellular redox couples (R/O) provide the electrons that are donated to DHA in Figure 4, Equation 1, in much the same way as previously described for the generic system presented in Figure 3. These redox couples either donate electrons directly or enzymatically, reducing DHA back to ascorbate (Linster and Van Schaftingen, 2007). Reduction of dehydroascorbate to ascorbate is facilitated within the cytosol by direct non-enzymatic reduction *via* glutathione (Winkler, 1992); by the NADPH-dependent enzymes thioredoxin reductase (May et al., 1997) or 3-alpha-Hydroxysteroid dehydrogenase (Del Bello et al., 1994); or through the gluthathione-dependent enzymes glutaredoxin (Wells et al., 1990), protein sulfide disomerase (Wells et al., 1990), and omega class glutathione transferase (Maellaro et al., 1997).

As outlined above, the release of ascorbate from the cell is facilitated via anion channels. Although the specific channels have not been identified for this particular tPMETS, it has been shown that physiologically this can occur using a variety of mechanisms. These include volume-sensitive and Ca^2+^-dependent anion channels (VSOAC), gap-junction hemichannels and exocytosis of secretory vesicles (Corti et al., 2010). Once released, direct interaction of ascorbate with ferric iron indicates that it mimics a ferri-reductase. This has further implications as it suggests this ascorbate-based cycling mechanism, in addition to an enzyme mediated transmembrane electron transfer mechanism (Figure 3), can be responsible for the reduction of ferric iron.

Lane et al. (Lane and Lawen, 2009) has previously highlighted the regulated release of intracellular ascorbate into the extracellular compartment, and discussed how this efflux constitutes an additional tPMETS. Their research indicated that this non-classical ascorbate export, which is achieved through the use of plasma membrane conduits and/or exocytosis, occurs as an alternative method of ascorbate-dependent tPMET. This is in contrast to the classical enzymatic electron transfer system, which uses the redox centers of plasma membrane ascorbate oxidoreductases such as Dcytb. Interestingly the paper suggests that the shuttling transport mechanism is not likely to be a general mechanism for ascorbate-stimulated iron reduction and that observations demonstrate that in a conditioned medium of ascorbate-supplemented cells a stimulation of ferricyanide reduction could not be induced despite a stimulation of ferric citrate reduction being possible through external ascorbate (May, 1999; Lane and Lawen, 2009, 2014). This is an important observation, which leads to the conclusion that there are two mechanisms used differentially for different iron compounds within the cited work. The physiologically relevant iron compound ferric citrate requires ascorbate to be effluxed from the cell for a stimulation of iron reduction to occur. In contrast, ascorbate can stimulate the reduction of non-physiological compounds (such as ferricyanide) by both ascorbate efflux mechanisms across the plasma membrane and “classical” enzymatic-based tPMET. In the same ascorbate-focused review of tPMET, Lane et al. (Lane and Lawen, 2009) also look at the possibility of the ascorbate free radical (AFR) in the reduction of extracellular iron, and there is evidence to suggest Dcytb can act as such.

Ascorbate has additionally been shown to have a function in the uptake of iron into astrocytes. Lane et al. demonstrated that when astrocytes were ascorbate-deficient, a yet unknown ferric iron uptake pathway predominates, but in ascorbate-replete systems both ferric and ferrous pathways are present. They state that in the ascorbate-replete system ferric iron is directly reduced via ascorbate, before transport into the cell via DMT1 (Lane et al., 2010). This further builds on the published work of Lane et al. on the role of ascorbate in ferrireduction and iron homeostasis.

Other more recent developments in this area include the work of Eccardt et al. (2017), who identify an ascorbate shuttling mechanism in skeletal muscle cells. It is clear that ascorbate is an important molecule for NTBI reduction and uptake within mammalian cells, but there are still questions to be answered on the exact mechanisms occurring.

## Biochemical control of electron transfer via tpmets

Whether it is by an enzyme dependent or independent pathway, the tPMETSs' function relies on the interaction of natural redox mediators. These are defined as molecules that are able to transfer electrons efficiently to a biocatalyst (Thévenot et al., 2001) and are intrinsic to the cells' ability to reduce extracellular iron. Consequently, the redox characteristics of the tPMETS must be considered to understand electron transport limitations controlling iron reduction and therefore transport via the ferri-reductase-DMT1 linked tPMET. Representing the external pool of iron to be transported via non-transferrin bound pathways that involve tPMETSs as “${\text{Fe}}_{\text{Ox}}^{\text{III}}$,” and the tPMETS as a whole as “Cell_red_” a simplified expression can be written for the overall reduction of ferric iron to ferrous iron (Equation 6). Although it is commonly assumed that the standard redox potentials of components of an electron transport chain must increase going from the initial electron donor to the final electron acceptor, the situation can be described by the Nernst equation for the system. For the redox reaction shown in Equation (6), the Nernst equation is given by Equation (7).

(1) $$\begin{array}{l} {\text{Fe}}_{\text{ox}+}^{\text{III}}{\text{Cell}}_{\text{red}}⇋{\text{Fe}}_{\text{red}+}^{\text{II}}{\text{Cell}}_{\text{ox}} \end{array}$$

where *E*_cell_ is the cell potential, $E_{\text{cell}}^{\theta }$ is the standard potential for the cell ($E_{\text{cell}}^{\theta }$ = *E*^θ^ (${\text{Fe}}_{\text{red}}^{\text{II}}$/${\text{Fe}}_{\text{Ox}}^{\text{III}}$/Fe) – *E*^0^(Cell_red_/Cell_ox_)), *R* = the gas constant (8.314 J K^−1^), *T* = temperature (assumed to be 298 K), *F* is Faraday's constant (96,487 C mol^−1^) and *n* = number of electrons transferred (assumed to be one).

(2) $$\begin{array}{l} {\text{E}}_{\text{cell}}\;=\;E_{\text{cell}}^{\theta }\;-\frac{RT}{nF}\;ln\;\frac{\left[(a{\text{Cell}}_{\text{ox}})][{\text{Fe}}_{\text{red}}^{\text{II}}\right]}{\;[{\text{Fe}}_{\text{ox}}^{\text{III}}][(a{\text{Cell}}_{\text{red}})]} \end{array}$$

At equilibrium, *E*^θ^_cell_ = 0, and hence the Nernst equation allows the position of the equilibrium for the reaction shown in Equation (6) to be determined. By considering the form of the Nernst equation, the following general comments can be made (based on thermodynamics only) pertinent to electron transfer from the cells to the external oxidized iron pool. When $E_{\text{cell}}^{\theta }>0$, electron transfer as represented by the forward direction in Equation (6) will not necessarily be complete, and conversely, even when $E_{\text{cell}}^{\theta }<0$ electron transfer may proceed to some extent in the forward direction. In other words, the extent of electron transfer from Cell_red_ (the tPMET) to ${\text{Fe}}_{\text{ox}}^{\text{III}}$ (the extracellular iron) depends on the concentration ratio of ${\text{Fe}}_{\text{ox}}^{\text{III}}$ and ${\text{Fe}}_{\text{red}}^{\text{II}}$ to cellular redox sites and also whether the reaction products are rapidly being re-converted to their initial forms to partake in the reaction again. In mammalian cells, the non-transferrin bound pathway has been reported to be more active, and operate more in situations where environmental iron is increased. It was stated that to deal with increases in extracellular iron, this upregulation of non-transferrin transport was required (Kaplan et al., 1991). Interestingly, no increased protein content was observed, and so an assertion was made that the reason for this increased transport via the non-transferrin pathways was as a result of a post translational modification to an already existing transporter. This means that the ratio of ${\text{Fe}}_{\text{ox}:}^{\text{III}}$ Cells_red_ would increase in situations where the extracellular NTBI levels rise and the equilibrium shown in Equation (6) would be pushed in the forward direction resulting in more iron being reduced when under thermodynamic control. Despite this observation others have noted that in iron deficient diets all cells or tissues upregulate DMT1 (Gunshin et al., 1997). In addition to this, the increased transport of ${\text{Fe}}_{\text{Ox}}^{\text{III}}$ via non-transferrin bound pathway may also occur due to local electronic effects, which increase transport through the DMT-1, which is discussed in the following section.

## DMT1 ion transport

At this point it is important to give more detail on the DMT1 transporter, namely the divalent metal transporting protein that forms the link with the tPMETSs that have been discussed, both transferrin and non-transferrin related. There is little known about the transporter DMT1, especially how DMT1 specifically targets divalent metals and the exact mechanisms by which transport occurs. DMT1 is also known as natural resistance-associated macrophage protein 2 (or Nramp2), and is encoded by the SLC11A2 gene. Mutation in the gene has been shown to play a role in such diseases as microcytic anemia and disease overload characteristics (Mims et al., 2005; Beaumont et al., 2006; Iolascon et al., 2006), indicating its role within iron transport. Despite being important for iron transport, DMT1 can also transport a host of other transition-metal ions, including copper, manganese, cobalt, cadmium, nickel, vanadium, and lead (Ehrnstorfer et al., 2014). DMT1 is ubiquitously expressed in all cell types, with its varying isoforms being expressed in different tissues (Soe-Lin et al., 2010). The different isoforms arise from splicing variants of the gene product, and variation in the structure of their 5′ and 3′ ends.

It has been demonstrated by Mackenzie et al. that all four isoforms function as metal-ion transporters of equivalent efficacy, with all managing to mediate ferrous iron uptake via a H^+^-coupled mechanism (Mackenzie et al., 2007). The authors indicated that its transport was controlled to an extent by the H^+^ inducing an electrochemical potential gradient. This was supported by Gunshin et al. who were one of the first to report on an insight into the mechanism by which transport is driven through DMT1 across the membrane (Gunshin et al., 1997). They stated that the process by which DMT1 mediates active transport of ferrous iron is voltage-dependent, and is coupled to the movement of H^+^ ions down their electrochemical gradient, into the cell. This was first proposed by Gunshin et al. (1997), but has also been confirmed by Mackenzie et al. in multiple publications (Mackenzie and Hediger, 2004; Mackenzie et al., 2006, 2007). They demonstrated that iron transport at low pH led to intracellular acidification, and with the pre-steady state currents observed during their electrochemical analysis, concluded that iron transport was pH dependent, thus indicating that H^+^ acts on the DMT1 to facilitate iron transport. In addition, they have also highlighted the importance of His-272 in this coupling process and suggested that protonation of this residue may be a requirement for H^+^-coupling (Mackenzie et al., 2006). The same conclusions have been drawn for the yeast homolog of DMT1, SMF1. Chen et al. demonstrated that when expressed in Xenopus oocytes, the SMF1 transporter exhibits proton-coupled metal cation transport but in addition, the transporter also uses proton-uncoupled transport of metal ions, not requiring H^+^ ions (Chen et al., 1999). Interestingly they also confirm the existence of the uncoupled “leak” pathway first suggested by Gunshin et al. Gunshin et al. also speculate that concomitantly with this path, the DMT1 acts as a H^+^ uniporter that “leaks” H^+^ ions into the intracellular compartment, which was confirmed by Mackenzie et al. (2006).

In addition, Mackenzie et al. also provided evidence that Fe^2+^ transport could also occur in an unlinked manner independent of protons, driven by the effect of membrane potential differences, albeit at a slower rate compared to coupled transport (Mackenzie et al., 2007). This suggests that the external pH and iron concentration is used as a means to facilitate and regulate the degree of non-transferrin dependent transport of iron.

**Figure 5:**
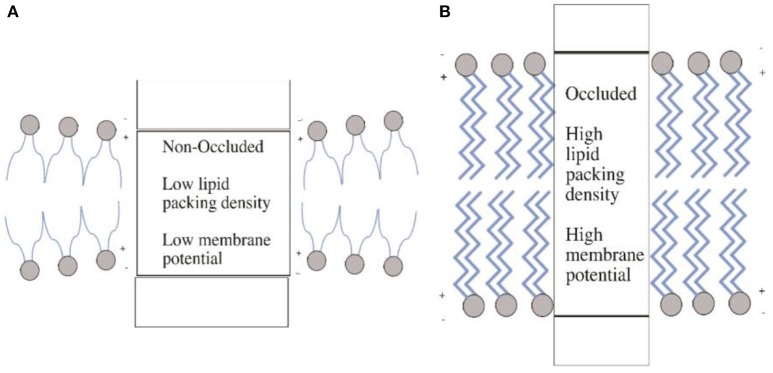
Membrane occlusion and intramembrane dipole potentials. **(A)** In the non-occluded state, the membrane is in a state of thinner (compared to **B**) hydrophobic thickness leading to a structural changes in the membrane protein. This in turn produces a low lipid packing density and thus low membrane potential. Panel **(B)** illustrates the opposite, where the occluded state leads to high packing density and thus high membrane potential. The increased hydrophobic thickness resulting from the occluded state causes these changes. Diagram adapted from Mares et al. (2014).

These data combined raise some interesting questions regarding the biochemical mechanisms that control the rate of iron transport via tPMET-DMT1 iron transport systems, which are yet to be solved. One of the most important is what aspect of ion transport through DMT1 is voltage-dependent. It was demonstrated that the co-transport of H^+^, which facilitates increased iron transport, was as a result of the protonation of a histidine within DMT1. It was suggested that this made the transport more thermodynamically favorable. However, Mares et al. (2014) have recently demonstrated that it not necessarily the binding of ions to a membrane extracellular transport site that is voltage dependent as classically thought (O'Shea, 2014), but is the occlusion of ions within the protein which is voltage dependent (Figure 5); occlusion being defined as the entrapment of an ion in a protein matrix. Binding ions such as H^+^ within DMT1, or an increase in charge interactions induced by changes in pH may affect the intramembrane dipole potentials. This in turn favors increased transport via changes in protein structure. These changes may be induced by voltage-geometric changes that occur within the membrane, which accompany transporter protein structural changes during occlusion and de-occlusion of the ion. Therefore, when considering the controlling mechanisms that determine transport through DMT1, it is important that this newly identified phenomenon is taken into consideration and further requires major experimental attention to determine the role that movement of charges across and within the membrane play in controlling iron transport via DMT-1.

## Other notable examples of membrane bound iron reducing systems

Ferrireduction does not necessarily require enzyme-mediation, or shuttling of reversible redox pairs. Prion diseases are neurodegenerative conditions in which the prion protein (PrP^C^) undergoes conformational changes to an aggregated, damaging form named PrP-scrapie (PrP^SC^). Haldar et al. have shown that the PrP^C^, using kidney proximal tubule (PT) cells, increases the uptake of NTBI and Tf-bound iron into the PT cells *in vivo*, and NTBI *in vitro* (Haldar et al., 2015). This is achieved by PrP^C^ acting as a ferri-reductase, which reduces iron thus making it more soluble to enable it to possibly be transported through transporters, and Tripathi et al. have since demonstrated that PrP^C^ acts as a ferri-reductase partner for both ZIP14 and DMT1 (Tripathi et al., 2015). Anion exchange has also been implicated in iron uptake in human bronchial epithelium cells by Ghio et al. (2003), where they showed the anion exchange protein 2 (AE2) facilitated the outward flux of superoxide for inward flux of bicarbonate, subsequently leading to superoxide mediated iron reduction.

In addition to the aforementioned routes of iron transport, there is another way iron can transverse the plasma membrane. This transport does not occur inwards, but outwards, via the protein ferroportin. Ferroportin or “solute carrier family 40 member 1” (SLC40A1) is the sole cellular efflux channel for iron, and is regulated by the hormone hepcidin, as discussed previously. The binding of hepcidin to ferriportin results in the internalization of the complex and subsequent degradation; meaning it is used when plasma iron levels are too high, or more iron is required within the cell (Donovan et al., 2005; Forman, 2005). Regulation of DMT1 and ferriportin, enables the flow of iron into and out of the cell to be carefully controlled.

Another review which covers the broader uses of tPMETs in general (not just the traditional models, and not just specifically relating to iron reduction) by Ly et al. may also be of use (Ly and Lawen, 2003). Another exceptional review regarding the broader context of mammalian tPMET and its implications for health and disease can be found by Del Principe et al. (2011).

In this review we have focused only upon the uptake of iron into mammalian cells, in substantial detail. A recent review published in Nature Reviews Drug Discovery by Crielaard et al. (2017) looks more broadly at iron homeostasis, with particular focus on targeting iron for drug discovery and delivery. It may be of interest to anyone reading this review who is seeking a broader overview of the topic.

## Conclusions

This review focuses upon the use of, and different mechanisms, of NTBI uptake, and more specifically the transplasma membrane transport (tPMET) that underpin the functionality of these systems. This review has sought to draw together previously unlinked data, namely how redox potential electrochemistry of the components of electron transfer lead to governance of biochemical control of tPMETSs, and how this interplays with NTBI transport on a macroscopic scale. We further seek to raise questions about how intramembrane dipole changes can lead to a membrane within an occluded or non-occluded state, and the implications for the transport of ions that this explains. In addition to fundamentally linking “micro” scale processes and biochemical control with “macro” scale transport of NTBI, we elaborate the wider field of iron homeostasis and the uptake of iron (not just NTBI) by cells by drawing conclusions about how tPMET is used differentially in different circumstances, and how this links to iron uptake. Transferrin-bound iron uptake is the predominate method of iron uptake within the body when in a healthy state. NTBI may exist up to 1 μg/ml in a healthy individual, although it is commonly undetectable, therefore although some NTBI uptake may occur this is very small in concern to overall iron levels. Therefore, in a healthy individual, it can be said that NTBI uptake occurs at a consequential level in two instances: at the lung epithelium where exposure to the atmosphere yields a higher exposure to iron, and at the gut epithelium (predominantly duodenum) where NTBI uptake is the main source of dietary adsorption of iron. Despite being used in certain instances in a healthy state, NTBI uptake systems seem to be present throughout the body. This leads to the explanation that NTBI uptake systems, that fundamentally employ tPMET to achieve this, are a safeguard against situations of iron overload and in disease states which may occur. We have brought together unlinked data that suggests tPMET not only plays a fundamental role in NTBI uptake in a state of disease where iron overload is present, but also that they may play a role when mitochondrial dysfunction occurs or where metabolic stress leads to an imbalance of NAD(P)H/NAD(P)^+^ levels resulting in increased NAD(P)H levels. This is where the cell uses tPMET to regenerate NAD(P)^+^ from NAD(P)H. This work should be of interest to the wider community of biology, because the work to date suggests a general mechanism of control that should be able to be applied to all cellular redox driven membrane transport systems. Similarly it should be of interest to the chemical community, which will in turn highlight the importance of how chemical systems modulate transport at molecular scale in the biological systems.

## Author contributions

HS, FR, AD, and KB contributed equally to writing the document. HS, FR, SS, AD, and KB edited the document. All authors read and approved the document for publication.

### Conflict of interest statement

The authors declare that the research was conducted in the absence of any commercial or financial relationships that could be construed as a potential conflict of interest.
